# Supplementary material for: Uncertainty-Aware Convolutional Neural Network for Identifying Bilateral Opacities on Chest X-rays: A Tool to Aid Diagnosis of Acute Respiratory Distress Syndrome
Source: Bioengineering (Basel). 2023 Aug 8;10(8):946. doi: 10.3390/bioengineering10080946 (PMC10451804; doi:10.3390/bioengineering10080946)
Supplement: Supplementary file 1 [file bioengineering-10-00946-s001.zip › bioengineering-2510253-supplementary.pdf]

bioengineering

Supplementary Materials

Uncertainty-Aware Convolutional Neural Network for Identifying Bilateral Opacities on Chest X-rays: A Tool to Aid Diagnosis of Acute Respiratory Distress Syndrome

Mehak Arora [1,2,*], Carolyn M. Davis [3,4], Niraj R. Gowda [5], Dennis G. Foster [3], Angana Mondal [2], Craig M. Coopersmith [3,4] and Rishikesan Kamaleswaran [2,4,*]

1 Department of Electrical and Computer Engineering, Georgia Institute of Technology, Atlanta, GA 30332, USA
2 Department of Biomedical Informatics, Emory University School of Medicine, Atlanta, GA 30332, USA; amonda3@emory.edu
3 Department of Surgery, Emory University School of Medicine, Atlanta, GA 30332, USA; carolyndavis@emory.edu (C.M.D.); dennis.gene.foster.iii@emory.edu (D.G.F.); cmcoop3@emory.edu (C.M.C.)
4 Emory Critical Care Center, Emory University School of Medicine, Atlanta, GA 30332, USA
5 Division of Pulmonary, Critical Care, Allergy and Sleep Medicine, Emory University School of Medicine, Atlanta, GA 30332, USA; niraj.raju.gowda@emory.edu
* Correspondence: marora42@gatech.edu (M.A.); rkamaleswaran@emory.edu (R.K.)

S1: Convolutional Neural Network Model Selection and Training Schemes

In our experiments, we adopted the DenseNet121 model architecture, which has proven to be a well-performing choice for multiple disease diagnosis on chest X-rays. Drawing from previous successful works, we applied histogram equalization and contrast enhancement as preprocessing steps to improve the diagnostic accuracy of our model[1]. Additionally, on-the-fly image transformations like random rotations, left-right flips, affine shifts, scaling, and random brightness and contrast adjustments were used for data augmentation during the training phase.

We experimented with a lung region segmentation pipeline, as proposed by Zhong et al. [2]. This pipeline achieved faithful segmentations of the lung region on most images in our dataset. However, a noteworthy observation was that the performance of our model decreased after using lung segmentation as a preprocessing step. Two primary reasons can be attributed to this outcome. Firstly, the chest X-ray images in our cohort were taken in the intensive care unit (ICU) and involved critically ill patients. Consequently, these images might often be of poor quality, containing occluding objects or being affected by suboptimal patient positioning. As a result, lung segmentations of such images could be inaccurate, leading to incorrect predictions by the model. Secondly, a key objective of our research was to train our model to predict the "equivocal" class accurately. However, this class may only be discernible by a physician when looking at the entire CXR image, rather than just a segmented lung region of interest (ROI). By applying lung segmentation as a preprocessing step, global context and information might be lost, making it more challenging for the model to correctly identify equivocal cases.

Results of Additional Experiments:

After a thorough review of recent works on CXR classification, we perform additional experiments as an attempt to improve the accuracy of our model. ResNets are widely acknowledged as state-of-the-art for image classification tasks [3] and have been frequently employed in medical image analysis. Recently, Vision Transformers have been shown to achieve superior performances [4] Nevertheless, it should be noted that Vision Transformers often demand substantial amounts of data for effective training. In contrast, the ConNeXt architecture, which is based on CNNs, has demonstrated superior accuracy

to Vision Transformers on the ImageNet dataset [5]. We train a ResNet34 and a ConvNeXt-tiny model on the three-class classification problem and report results in **Table S1**. We subsequently build an ensemble classifier by averaging the predicted probability of all three models. It is noticeable that the DenseNet121 model shows superior performance to other individual models. However, the ensemble model shows a higher AUROC, AUPRC, specificity, and diagnostic odds ratio.

Table S1. Performance Metrics of the Three Class Model (predicting "present", "absent" and "equivocal"). The highest values for each metric are emboldened. 95% Confidence Intervals are reported and were calculated using non-parametric estimation using bootstrapping.

Model	AUROC	AUPRC	Fscore	Precision	Sensitivity	Specificity	Diagnostic Odds Ratio
Resnet-34	0.796 (0.769, 0.826)	0.879 (0.865, 0.887)	0.716 (0.688, 0.742)	0.712 (0.684, 0.742)	0.729 (0.701,0.761)	0.704 (0.652, 0.757)	6.390 (4.427, 9.821)
ConvNeXt-tiny	0.824 (0.800, 0.850)	0.907 (0.821, 0.916)	0.709 (0.679, 0.737)	0.716 (0.690, 0.744)	0.743 (0.718, 0.770)	0.820 (0.778, 0.859)	13.134 (9.134, 19.865)
DenseNet	0.828 (0.803, 0.853)	0.874 (0.861, 0.887)	**0.746 (0.720,0.775)**	**0.755(0.731, 0.782)**	0.761 (0.735, 0.786)	0.842 (0.812, 0.875)	17.211 (12.106, 25.976)
Ensemble Model	**0.854 (0.825, 0.876)**	**0.920 (0.909, 0.931)**	0.716 (0.685, 0.734)	0.749 (0.729, 0.769)	**0.771 (0.739, 0.798)**	**0.950 (0.921, 0.978)**	**35.121 (22.561, 45.891)**

S2: Cross-Validation Performance

We employed the bootstrap method to calculate non-parametric confidence intervals on our results and report all performance metrics with the 95% confidence interval. This accounts for variance in the data distribution. As suggested by the reviewer, we present the results of a 10-fold cross-validation on the internal test set in **Table S2**, along with the mean and standard deviation for each metric.

Table S2. Results of 10-fold cross-validation using the DenseNet121 model trained with uncertainty-aware cross-entropy loss with probability targets.

Cross-Validation Fold	AUROC	AUPRC	F-score	Precision	Sensitivity	Specificity	Diagnostic Odds Ratio	Balanced Accuracy
Fold-1	0.828	0.874	0.746	0.755	0.761	0.842	17.211	0.802
Fold-2	0.829	0.891	0.749	0.746	0.764	0.774	11.055	0.769
Fold-3	0.789	0.844	0.781	0.788	0.660	0.775	5.215	0.718
Fold-4	0.772	0.852	0.678	0.687	0.698	0.775	7.955	0.737
Fold-5	0.822	0.885	0.679	0.720	0.727	0.898	23.395	0.813
Fold-6	0.815	0.857	0.741	0.739	0.743	0.716	5.215	0.729
Fold-7	0.801	0.881	0.721	0.719	0.737	0.730	7.642	0.733
Fold-8	0.851	0.907	0.772	0.769	0.787	0.730	15.782	0.759
Fold-9	0.829	0.843	0.685	0.726	0.748	0.924	35.921	0.836
Fold-10	0.804	0.876	0.656	0.703	0.724	0.893	22.435	0.809
**Mean**	**0.814**	**0.871**	**0.721**	**0.735**	**0.735**	**0.806**	**15.183**	**0.770**
**Std. dev.**	**0.023**	**0.021**	**0.044**	**0.031**	**0.036**	**0.077**	**9.885**	**0.041**

References:

1. Nasser, A.A.; Akhloufi, M.A. A Review of Recent Advances in Deep Learning Models for Chest Disease Detection Using Radiography. *Diagnostics* **2023**, *13*, 159. https://doi.org/10.3390/diagnostics13010159.
2. Zhong, A.; Li, X.; Wu, D.; Ren, H.; Kim, K.; Kim, Y.; Buch, V.; Neumark, N.; Bizzo, B.; Tak, W.Y.; et al. Deep metric learning-based image retrieval system for chest radiograph and its clinical applications in COVID-19. *Med. Image Anal*. **2021**, *70*, 101993. https://doi.org/10.1016/j.media.2021.101993.
3. He, K.; Zhang, X.; Ren, S.; Sun, J. Deep residual learning for image recognition. In Proceedings of the IEEE Conference on Computer Vision and Pattern Recognition 2016, Las Vegas, NV, USA, 27–30 June 2016.
4. Dosovitskiy, A.; Beyer, L.; Kolesnikov, A.; Weissenborn, D.; Zhai, X.; Unterthiner, T.; Dehghani, M.; Minderer, M.; Heigold, G.; Gelly, S.; Uszkoreit, J. An image is worth 16x16 words: Transformers for image recognition at scale. *arXiv* **2020**, *arXiv*: 2010.11929.
5. Liu, Z.; Mao, H.; Wu, C.Y.; Feichtenhofer, C.; Darrell, T.; Xie, S. A ConvNet for the 2020s. In Proceedings of the IEEE/CVF Conference on Computer Vision and Pattern Recognition (CVPR), New Orleans, LA, USA, 18–24 June 2022.
